# A Clinical Audit of “Negative Symptoms” Within an Assertive Outreach and Rehabilitation (AORT) Process

**DOI:** 10.1192/bjo.2026.11452

**Published:** 2026-06-30

**Authors:** Omowumi Oso, Bethany Stubbs, Clementine Edwards, Isaac Akande

**Affiliations:** 1 Herefordshire and Worcestershire Health and Care Trust, Worcester, United Kingdom; 2 Canterbury Christ Church University & Surrey and Borders Partnership NHS, Surrey, United Kingdom; 3 Kings College London, London, United Kingdom; 4 South London and Maudsley NHS Foundation Trust, London, United Kingdom

## Abstract

**Aims:**

Loss of emotional experience and expression, known as “negative symptoms”, can significantly impact people’s emotional, cognitive, and psychological functioning. However, available evidence suggests these symptoms are often missed due to overshadowing by other difficulties. Consequently, there is a dearth of evidence for effective management. The lack of prevalence data challenges treatment resource allocation within healthcare services. Thus, this audit aims to collect relevant data on negative symptoms among service users in an NHS Trust.

The aim of this project is to understand the prevalence, demographic profile and description of “negative symptoms” among a sample of AORT service users. It also investigates the allocation of resources for its management.

**Methods:**

This is a mixed methods study. 100 service users who were open to AORT were randomly selected between 1 September and 31 November 2023 through the clinical record interactive search service (CRIS). Data relating to the demographics of the participants, their psychological and occupational therapy contact, and prescribed medications were extracted and anonymised. Two independent coders also read through the electronic patient journey system (ePJS) to record the language used to describe “negative symptoms” and identify occupational therapy contact.

**Results:**

Findings revealed that only 28% of the service users reported “negative symptoms”, with most of the reports among Black/Black British–Caribbean males. Among the study sample, only half were prescribed medication, and people with “negative symptoms” were less likely to receive occupational or psychological therapies than those without. However, no significant associations with age, gender, or race/ethnicity were found.

**Conclusion:**

Low prevalence of therapeutic contacts among service users who report “negative symptoms” may be related to multiple factors such as complex diagnostic tools and priorities highlighted in management pathways. These factors may increase the difficulties of expressing service users’ needs, leading to a vicious feedback loop that may become complicated to manage without support.

Next steps: A better diagnostic process could uplift the “negative symptoms” management and resource allocation. This could be achieved through a co-learning workshop on topics such as the clinical presentation and impact of “negative symptoms” and co-creation of simpler assessment proformas. It is also important that “negative symptoms” are considered in risk assessments. Finally, peer support and psychological therapeutic groups can be facilitated by teams to improve the management of “negative symptoms”.

